# Effectiveness of a Nutritional Mobile Application for Management of Hyperphosphatemia in Patients on Hemodialysis: A Multicenter Open-Label Randomized Clinical Trial

**DOI:** 10.3390/jpm12060961

**Published:** 2022-06-12

**Authors:** Lee-Fang Teong, Ban-Hock Khor, Hi-Ming Ng, Sharmela Sahathevan, Kristo Radion Purba, Sreelakshmi Sankara Narayanan, Abdul Halim Abdul Gafor, Bak-Leong Goh, Boon-Cheak Bee, Rosnawati Yahya, Sunita Bavanandan, Zaimi Wahab, Sadanah Aqashiah Mazlan, Karuthan Chinna, Zaki Morad, Zulfitri Azuan Mat Daud, Tilakavati Karupaiah

**Affiliations:** 1 School of Biosciences, Faculty of Health and Medical Sciences, Taylor’s University, Subang Jaya 47500, Malaysia; teongleefang@gmail.com (L.-F.T.); sreelakshmiprem@hotmail.com (S.S.N.); 2 Department of Dietetics and Food Service, Selayang Hospital, Batu Caves 68100, Malaysia; 3 Faculty of Food Science and Nutrition, Universiti Malaysia Sabah, Kota Kinabalu 88400, Malaysia; khorbanhock@gmail.com; 4 Department of Dietetics & Nutrition Services, Sunway Medical Center, Petaling Jaya 47500, Malaysia; nghiming@gmail.com; 5 Department of Allied Health Sciences, Faculty of Science, Universiti Tunku Abdul Rahman, Kampar 31900, Malaysia; sham_0901@yahoo.com; 6 School of Computer Science, University of Southampton Malaysia, Iskandar Puteri 79100, Malaysia; kr.purba@soton.ac.uk; 7 Department of Medicine, Faculty of Medicine, Universiti Kebangsaan Malaysia Medical Center, Kuala Lumpur 56000, Malaysia; halimgafor@gmail.com; 8 Clinical Research Center, Serdang Hospital, Kajang 43000, Malaysia; bak.leong@gmail.com; 9 Department of Nephrology, Selayang Hospital, Lebuh Raya Selayang-Kepong, Batu Caves 68100, Malaysia; drbeebc@gmail.com; 10 Department of Nephrology, Kuala Lumpur Hospital, Jalan Pahang, Kuala Lumpur 53000, Malaysia; rosnayahya@gmail.com (R.Y.); sbavanandan@gmail.com (S.B.); zaimi.wahab@gmail.com (Z.W.); 11 Department of Medicine, Kajang Hospital, Jalan Semenyih, Kajang 43000, Malaysia; sadanahmazlan@gmail.com; 12 Faculty of Business and Management, USCI University, Cheras, Kuala Lumpur 56000, Malaysia; karuthan@gmail.com; 13 National Kidney Foundation Malaysia, Petaling Jaya 46100, Malaysia; zakimorad@gmail.com; 14 Department of Dietetics, Faculty of Medicine and Health Sciences, Universiti Putra Malaysia, Serdang 43400, Malaysia; zulfitri@upm.edu.my

**Keywords:** mobile app, nutrition education, hemodialysis, hyperphosphatemia, phosphorus, patient-centered care

## Abstract

This study aims to determine the effectiveness of a phosphate mobile app (PMA), MyKidneyDiet-Phosphate Tracker ©2019, on hemodialysis (HD) patients with hyperphosphatemia. A multicenter, open-label, randomized controlled trial design allowed randomization of patients with hyperphosphatemia to either the usual care group (UG; receiving a single dietitian-led session with an education booklet) or the PMA group (PG). Thirty-three patients in each intervention group completed the 12-week study. Post-intervention, serum phosphorus levels were reduced in both groups (PG: −0.25 ± 0.42 mmol/L, *p* = 0.001; UG: −0.23 ± 0.33 mmol/L, *p* < 0.001) without any treatment difference (*p* > 0.05). Patients in both groups increased their phosphate knowledge (PG: 2.18 ± 3.40, *p* = 0.001; UG: 2.50 ± 4.50, *p* = 0.003), without any treatment difference (*p* > 0.05). Dietary phosphorus intake of both groups was reduced (PG: −188.1 ± 161.3 mg/d, *p* < 0.001; UG: −266.0 ± 193.3 mg/d, *p* < 0.001), without any treatment difference (*p* > 0.05). The serum calcium levels of patients in the UG group increased significantly (0.09 ± 0.20 mmol/L, *p* = 0.013) but not for the PG group (−0.03 ± 0.13 mmol/L, *p* = 0.386), and the treatment difference was significant (*p* = 0.007). As per phosphate binder adherence, both groups reported a significant increase in Morisky Medication Adherence Scale scores (PG: 1.1 ± 1.2, *p* < 0.001; UGa: 0.8 ± 1.5, *p* = 0.007), without any treatment difference (*p* > 0.05). HD patients with hyperphosphatemia using the PMA achieved reductions in serum phosphorus levels and dietary phosphorus intakes along with improved phosphate knowledge and phosphate binder adherence that were not significantly different from a one-off dietitian intervention. However, binder dose adjustment with meal phosphate content facilitated by the PMA allowed stability of corrected calcium levels, which was not attained by UC patients whose binder dose was fixed.

## 1. Introduction

A significant proportion of patients with chronic kidney disease (CKD) progressively develop CKD–mineral bone disorder (CKD–MBD), with hyperphosphatemia as a major causative factor, particularly when reaching end-stage kidney disease (ESKD), which requires dialysis [1,2]. The serum phosphorus abnormality increases the risk of bone disorders, hip fractures, secondary hyperparathyroidism, vascular calcification, cardiovascular diseases, and mortality [3,4].

Alongside dialysis removal of serum phosphate, dietary phosphorus restriction and the use of phosphate-binding medications are crucial co-strategies for the management of hyperphosphatemia [2]. However, most foods high in phosphorus are also high in protein, and restriction of dietary phosphorus could potentiate a concomitant suboptimal dietary protein intake (DPI) [5]. The accompanying metabolic sequelae would be a negative nitrogen balance, gluconeogenesis, and muscle tissue degradation, given the background of protein and amino acid losses during the dialysis procedure [6]. Therefore, relying on limiting dietary phosphorus to control hyperphosphatemia is not feasible, considering that dialysis patients have a higher protein requirement [7]. Although the use of phosphate binders does allow a more liberal protein intake for dialysis patients, the current practice of a fixed phosphate dosing prescription without titrating to dietary phosphorus intake promotes a greater risk of vascular calcification from the overdosing of calcium-based phosphate binders [2], which are the lowest cost option for many low- to middle-income countries [8].

Successful management of hyperphosphatemia calls for the integrated management of concerned healthcare professionals such as dietitians (delivering nutritional education related to dietary phosphorus) and physicians (prescribing and adjusting the phosphate binder dose based on food phosphorus content) [9]. These two patient-dependent strategies are vulnerable when the caregivers’ advice to patients is not coordinated. Indeed, the Global Kidney Nutrition Care survey reveals that dietitians and nephrologists communicated only ‘sometimes’ in 60% of the 155 surveyed countries. This survey further notes that patient access to dietitian counseling was most unsatisfactory (only 48% of 155 countries) and absent in 23% of low-income countries [10]. This is not surprising as an earlier *Global Nephrology* survey conducted by the International Society of Nephrology identified that dietitians were the largest group (78%) in the shortage of healthcare professionals required to enable a sustainable kidney care system [11].

A challenging issue in promoting desirable behaviors to achieve optimal serum phosphorus control via regulating a patient’s dietary phosphorus intake is that the food intake patterns of patients are complex [12]. Well-cited barriers in the context of patients include lack of knowledge and motivation, lack of adherence to a low phosphate diet, and low compliance to phosphate binders [13]. The mode of delivery of patient education, which is dependent on its didactic content, may be to blame, along with limited dietitian access to provide personalized counseling and the exclusion of binder management from the patient education provided by the dietitian.

The Malaysian scenario of hyperphosphatemia management typifies a low resource setting, with no dietitian access and a lack of calibrating binder doses with dietary phosphorus consumption. These issues were acknowledged in a nationwide survey on the state of nutrition care in outpatient HD settings in 2018 [14]. It was noted that non-dietitian healthcare professionals, such as doctors and nurses, were primarily delivering standard patient education on nutrition. The need to encourage protein consumption in Malaysian HD patients is also vital, as separate studies have noted inadequate nutritional intake and malnutrition [15], especially protein–energy wasting with evidence of muscle wasting been prevalent [16].

Given these issues, we sought a novel approach to promoting interprofessional care, which is critical to the integrated care model to enable sustainable kidney nutrition care, especially in countries with limited, insufficient, or lack of access to dietitians. Partnering with other healthcare professionals within a team may overcome a shortfall in patient care because of a missing or limited service from a professional [17]. A mobile app focusing on phosphate management, specifically the phosphate mobile app (PMA), can be an innovative approach to narrowing the gaps in phosphate education delivery to CKD patients. We had earlier conceptualized and developed a PMA-triangulating meal dietary phosphorus content assessment with binder dose prescription, which was validated by experts before been tested for acceptance by HD patients [18]. The mobile app received high acceptability from HD patients. However, the efficacy of this mobile app’s implementation in clinical settings for hyperphosphatemia management has not been evaluated. This study, therefore, purposively carried out this objective through an open-label, multicenter, randomized clinical trial, where a usual care model with a dietitian was used as a comparator treatment.

## 2. Materials and Methods

### 2.1. Study Design and Subject Enrollment

This was an open-label, multicenter, randomized clinical trial with ethical approval received from both the Human Ethics Committee of Taylor’s University (HEC 2019/011) and the Medical Research Ethics Committee of the Ministry of Health, Malaysia (NMRR-19-3825-45381). This study was registered with ClinicalTrials.gov (ID: NCT04789876).

Eligible adult patients (≥18 years old) receiving maintenance HD treatment in Klang Valley, Malaysia, were enrolled into the study from November 2019 to February 2020. The inclusion criteria were patients receiving standard HD treatment (thrice-weekly, four hours per session), dialysis vintage ≥ three months, serum phosphorus level above 1.78 mmol/L for the past three months, Kt/V ≥ 1.2, literate for the English, Malay, or Mandarin languages, owning a smartphone, able to use at least one comprehensive mobile application independently, with internet access, able to perform self-care and be independent, and willing to adhere to all study requirements and protocols. We excluded patients who were on hemodiafiltration, multiple types of phosphate binder prescriptions, receiving phosphate education from a dietitian over the past one year, had a history of hospitalization within three months prior to recruitment to this study, had undergone a parathyroidectomy, were terminally ill, residing in institutionalized settings, with visual or cognitive impairment, and/or had dementia or developmental delays. Patients on multiple phosphate binders were excluded because the phosphate algorithm of the PMA did not support a combination of different phosphate binders. Single phosphate binder prescription is the norm in Malaysia [19].

The sample size was determined using the á *priori* power analysis generated by the G*Power 3.1.9.6, Franz Faul (Universitat Kiel, Germany) software calculator based on a 12-week serum phosphorus mean change, as reported for a randomized controlled trial study related to phosphate education [20].

### 2.2. Randomization

After signing informed consent forms, patients were randomized into two study groups using an online research randomizer tool (https://www.randomizer.org/, accessed on 11 October 2019). The randomization process used a cluster-randomized approach based on the prevailing dialysis session shifts at HD centers to avoid allocating patients within the same shift to different treatments. An independent third-party coin toss was used to determine the type of intervention for each shift group.

### 2.3. Study Groups

Recruited patients for the two study groups underwent 12 weeks of intervention as per their allocated treatment plan. All patients received the usual medical care from their respective HD centers during this period, apart from the intervention treatment, as detailed below:

*Usual Care Group (UG)*—The comparator treatment group was UG patients who received a single nutrition counseling session with a dietitian on hyperphosphatemia management at baseline, with the session lasting 30 to 40 min. Topics covered basic knowledge on hyperphosphatemia, dialysis removal of phosphorus, sources of dietary phosphorus (e.g., animal, plant, and inorganic) with examples of foods high and low in phosphorus content, and the use of phosphate binders. All these topics were also accessible to the patient in a 12-page illustrated booklet available in the English, Malay, and Mandarin languages.

*PMA Group (PG)*—PG patients received access to the PMA, ©2019 *MyKidneyDiet-Phosphate Tracker.* The development process and features of this PMA have been previously described [18]. In brief, the PMA carries six animated education videos covering topics on hyperphosphatemia, dialysis, phosphate binders, dietary phosphorus, lifestyles, and the responsibilities of a dialysis patient. Importantly, the PMA carries an interactive food database of more than 500 foods commonly consumed by HD patients living in Klang Valley, Malaysia, and functions as a personalized diet calculator. Its interactive database enables meal calculations for energy, protein, sodium, potassium, and phosphorus, and the required phosphate binder dose is titrated to the phosphate content of foods chosen for the meal by the patient. The PMA is also available in three languages, namely, English, Malay, and Mandarin. PG patients were assisted with the PMA’s installation and familiarized with all the features of the PMA, particularly engaging with the food database to enable meal phosphorus content calculation and titration to the phosphate binder dose. All patients were required to register an account for this PMA using their phone number, which allowed cross-checking, with permission at the back-end server of the PMA for compliance monitoring.

There was no active follow-up during the 12-week intervention period. Therefore, both groups underwent three encounters with the researcher in total, namely, screening for eligibility and obtaining informed consent, baseline data assessment and access to education either with the dietitian or the PMA, and post-assessment after 12 weeks.

### 2.4. Outcome Measures

We assessed serum phosphorus as the primary outcome for this study, whilst the secondary outcomes included other biochemical parameters related to CKD–MBD, dietary phosphorus intake, patients’ knowledge of phosphorus management, and phosphate binder adherence. All outcomes were measured at baseline before interventions were provided and after completion of the 12-week intervention period.

#### 2.4.1. Biochemical Parameters

Patient data for serum phosphorus, corrected calcium, intact parathyroid hormone (iPTH), alkaline phosphatase (ALP), and albumin were obtained from routine laboratory investigations performed at their respective dialysis centers. 

#### 2.4.2. Patients’ Knowledge of Phosphorus Management

An expert-validated phosphate knowledge questionnaire, benchmarked to clinical practice guidelines [7] and culturally appropriate to the local context [15], was utilized to assess knowledge of phosphorus management for both treatment groups. This questionnaire consisting of 18 questions assessed overall phosphate management (3 items), consequences of hyperphosphatemia (3 items), phosphate binders (4 items), dietary phosphorus (7 items), and responsibility (1 item). Each question offered multiple choice answers with only one best possible choice, inclusive of a “not sure” option. Each correct answer was allocated a score of 1, with a maximum total score of 18. The questionnaire was available in the English, Malay, and Mandarin languages.

#### 2.4.3. Dietary Intake

Patients’ dietary intake was assessed using the 3-day dietary recall (3DDR) method, inclusive of a dialysis day, a non-dialysis day, and a weekend day [7]. The 3DDR was collected via face-to-face interviews of patients with the dietitian. Standard household tools were used to optimize the recall of portion sizes of the food and beverages consumed. Intakes were recorded in household units, which were then converted to weight in grams. All nutritional analysis was performed using Nutritionist Pro^TM^ 2.2.16 First Databank software (Axxya System LLC, Stanford, TX, USA), which references the Malaysian Food Composition [21] and Singapore Food Composition databases [22]. The Goldberg cut-off was applied to identify the reliability of dietary data recall based on the Energy Intake:Basal Metabolic Rate (EI:BMR) index using the Harris–Benedict formula [23]. Values <0.8 were classified as under-reporting, and values of 0.8–2.4 were considered acceptable.

#### 2.4.4. Phosphate Binder Adherence

Patients’ adherence to their prescribed phosphate binder was assessed using the Morisky Medication Adherence Scale (MMAS-4) [24]. MMAS-4 is a validated and sensitive 4-item self-reporting measure to assess medication-taking behaviors over the past week of assessment. The scoring scheme of MMAS-4 with “Yes” = 0 and “No” = 1 for each item, yields a total score ranging from 0 to 4.

#### 2.4.5. Sociodemographic and Medical History

We collected patients’ sociodemographic data for age, gender, ethnicity, marital status, education level, employment status, and monthly income. Patients’ medical history, including first dialysis initiation date, Kt/V, medication prescriptions, underlying and previous medical diagnoses, present and past hospitalizations and treatments, were extracted from their medical records. Anthropometric measurements pertaining to weight, as per pre-HD, post-HD, and dry weight status, were obtained from patients’ dialysis logbooks. Patients were asked about their bowel pattern and chewing ability to rule out factors contributing to poor phosphate clearance.

### 2.5. Statistical Analysis

The Shapiro–Wilk test was used to assess the normality distribution of continuous variables. Continuous variables were presented as mean ± standard deviation for variables with normal distribution or as median (interquartile range (IQR)) for skewed variables. Categorical variables were expressed as frequency (percentages). Independent *t*-tests and Mann–Whitney tests were applied to compare mean and mean-rank differences, respectively, for the baseline characteristics between UG and PG. Pearson’s chi-squared test or Fisher’s exact test was used as appropriate, to determine the associations between two categorical variables. Paired-samples *t*-tests were applied to determine the changes of normally distributed variables at baseline and the end of 12 weeks, whilst Wilcoxon’s signed-rank tests determined the median differences for skewed variables.

Change in outcome measures between the intervention groups were compared using independent *t*-tests or Mann–Whitney tests, as appropriate. The 95% confidence interval (CI) of the mean difference is reported. For normally distributed variables, Cohen’s *d* value with 95% CI was used to determine the effect size of the mean difference, and the effect size was interpreted as small (*r* = 0.10), medium/moderate (*r* = 0.30), and large (*r* = 0.50). For the 3DDRs, 17 patients (26%) were excluded from the analysis due to under-reported and over-reported dietary intakes.

All statistical analyses were performed using IBM SPSS Version 26 (IBM SPSS Statistics Inc. Chicago, IL, USA), and statistical significance was set as *p* < 0.05 for all evaluated parameters.

## 3. Results

### 3.1. Baseline Characteristics

**Figure 1:**
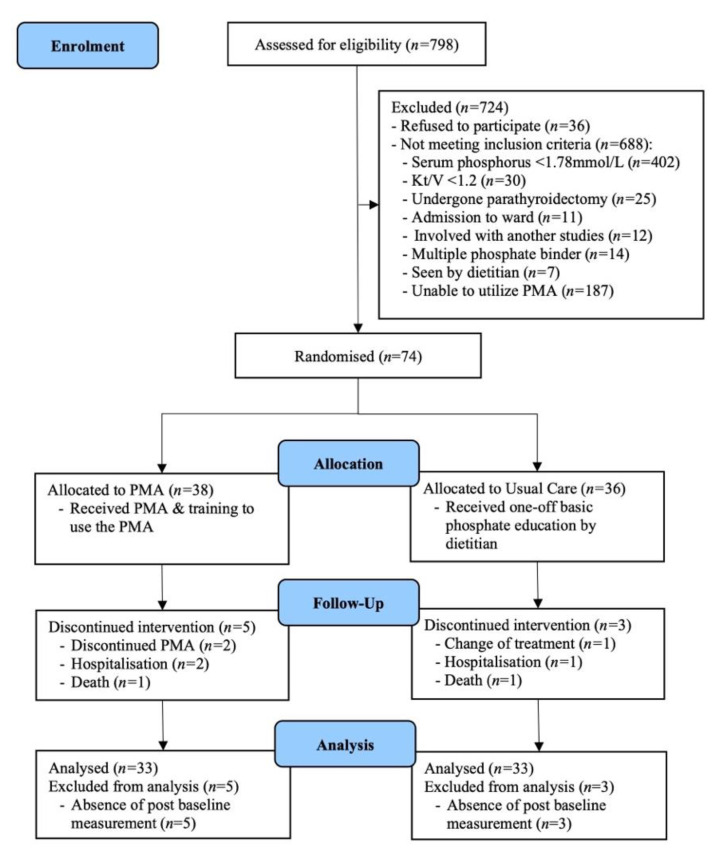
Flow diagram of the study. Abbreviation: PMA, phosphate mobile application.

Out of 74 consenting patients enrolled into this study, 66 patients (89.2%) completed the study with 33 patients for each group. Mortality incidence was similar for both groups (*n* = 1 per group), whilst three PG patients withdrawing from the study. Other reasons for patient discontinuation included hospitalization and change of treatment modality (Figure 1). Patient characteristics and medical history at baseline are presented in Table 1, and all comparators were homogenous between groups (*p* > 0.05). Of importance, the mean (± SD) age of patients was 48.3 ± 14.4 years, and the majority were female (53%) and Malay (50%). The median (IQR) for dialysis vintage was 67.8 (69.8) months. Most patients were on calcium carbonate (86%), followed by sevelamer carbonate (8%) and lanthanum carbonate (6%). The Android operating system was the most common operating system (92.4%) in patients’ smartphones.

### 3.2. Biochemical Parameters

**Figure 2:**
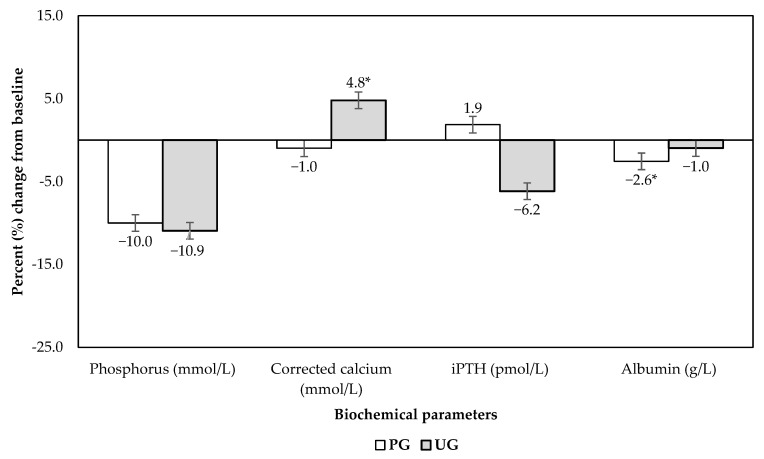
Percent change in biochemical parameters from baseline. * *p* < 0.05. Abbreviation: PG, PMA group; iPTH, intact parathyroid hormone; UG, usual care group.

Baseline and end-of-12-weeks values of biochemical parameters for UG and PG patients are presented in Table 2, and percent change data corrected to baseline values are presented in Figure 2. Serum phosphorus levels as the primary outcome from the 12-week intervention were not significant between treatments (*p* > 0.05), indicating that both the PMA and the standard dietitian session supported by the dietary phosphorus education booklet produced equivalent significant reductions within groups (both *p* = 0.001), with the quantum of reduction being 10–11%. After 12 weeks, 8 and 14 patients, as per PG and UG interventions, respectively, had their serum phosphorus levels fall within the normal range (<1.78 mmol/L). The corrected calcium levels significantly increased by 4.8% for UG patients (*p* = 0.013), whilst levels for PG patients remained unchanged (*p* > 0.05), resulting in significant percent change differences between groups (*p* = 0.011). UG patients also experienced a concomitant 6.2% reduction in iPTH, although this was non-significant. Changes in serum albumin and total protein levels were not significantly different between the two groups, although a significant reduction of serum albumin was noted within PG.

### 3.3. Knowledge Score, Phosphate Binder Compliance, and Dietary Intakes

Tracking change data, relating to patients’ knowledge scores, MMAS-4 scores, and dietary intakes for both groups, are shown in Table 3. Imparting phosphorus education either by the single dietitian session or the PMA carried equivalent impacts on overall knowledge scores between groups post-intervention (*p* > 0.05), with each treatment attaining significance within groups, with a large effect size (mean Δ = 2.2 ± 3.4, *d* = 0.64, *p* = 0.001 for PG vs. 2.5 ± 4.5, *d* = 0.56, *p* = 0.003 for UG).

Patients’ adherence to their prescribed phosphate binder, as indicated by their MMAS-4 scores, improved from baseline for both groups, whilst the between-group difference was not statistically significant (*p* > 0.05). The medication adherence of PG increased from 12.1% to 60.6% post-intervention, with a 48.5% improvement, whilst for UG, the medication adherence increased from 15.2% to 48.5% post-intervention, with a 33.3% improvement.

**Figure 3:**
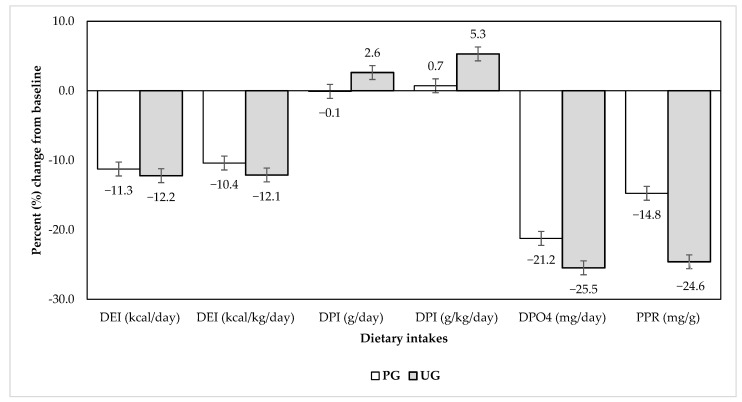
Percent change in dietary intake; *p*-values for all comparisons were >0.05. Abbreviation: DEI, dietary energy intake; DPI, dietary protein intake; DPO4, dietary phosphorus intake; PG, PMA group; PPR, phosphorus-to-protein ratio; UG, usual care group.

The energy, protein, phosphate, and phosphorus-to-protein ratio (PPR) profiles of the consumed diets, assessed at the end of 12 weeks, were not significantly different between both groups (*p* > 0.05). However, within-group reductions in intakes of dietary energy, phosphate, and PPR occurred for both groups, with the largest effect size related to dietary phosphorus (both *d* = 1.17) and PPR (mean Δ = −1.9 ± 3.1, *d* = 0.62 for PG, and mean Δ = −3.7 ± 3.5, *d* = 1.07 for UG). The percent changes for dietary intakes from baseline are presented in Figure 3.

## 4. Discussion

A recent meta-analysis of clinical trials revealed that diet therapy provided by a dietitian was effective in lowering serum phosphorus levels in HD patients with hyperphosphatemia, though the overall quality of the evidence was low [25]. However, evidence for the effectiveness of a nutrition mobile app for the management of hyperphosphatemia remains inconclusive. A single-arm study by El-Khoury et al. [26] showed no significant difference in serum phosphorus levels among HD patients after using an education and self-monitoring mobile app (KELA.AE) for 2 weeks, despite an improvement in knowledge about phosphorus being observed. This finding could be attributed to the relatively short study duration. Another study by Farfan-Ruiz et al. [27] demonstrated that patients on peritoneal dialysis, assigned to either a usual care group or a mobile app (OkKidney app) group, did not experience significant reductions in serum phosphorus levels after three months. Similarly, Chiang et al. [28] showed that the post-3-month serum phosphorus levels of HD patients using a phosphate control smartphone application were not significantly different from patients receiving usual care, although some users did experience a decrease in serum phosphorus levels. Contrary to these studies, our findings show both educational interventions, using either the PMA or one-off face-to-face dietitian counseling using didactic education material, were equally effective in reducing serum phosphate levels significantly in HD patients after 12 weeks of intervention. Our study’s intervention with the mobile app indicated a positive impact, in contrast to the other studies. This is mainly attributed to the intervention tools (the mobile app and educational pamphlets) being validated by multi-disciplinary experts and undergoing testing for acceptance by HD patients [18]. Secondly, the study was conducted at multiple centers for 3 months with systematic randomization, and analyzed using modified intention-to-treat protocols, whilst the other studies were executed as pilot trials.

Beyond the primary outcome of serum phosphate levels, we also observed a significant treatment effect on serum-corrected calcium (SCC) levels associated only with UG patients. The increase in SCC levels for UG was accompanied by a twice-greater non-significant reduction in iPTH levels compared to PG patients, which could be attributed to improved adherence to the calcium-based phosphate binders. Indeed, about 87.9% of UG patients were prescribed these phosphate binders, and they were probably diligently complying with the nutrition education provided by the dietitian. Similar improved adherence to the phosphate binder prescription, as assessed by the Morisky scale, was noted for both groups. The practice of fixed phosphate binder dosing and dietary phosphorus intake mismatch leads to the excessive consumption of calcium-based phosphate binders, which increases the risk for hypercalcemia [29]. Hypercalcemia is associated with vascular disease and cardiovascular mortality [30].

Assessments of patient dietary phosphorus and protein intakes and calculating dietary PPRs are critical to the study’s primary objective of hyperphosphatemia management. We observed significant reductions of more than ~20% in dietary phosphorus intakes for both groups. Despite protein food sources being the prime source of phosphorus, the post-3-months dietary protein intakes remained consistent with baseline values for both groups, suggesting that the dietary phosphorus reduction was likely due to the reduced consumption of non-protein sources of phosphorus, such as carbonated beverages and sugar-sweetened beverages, which are common in this patient population [31]. In tandem, the PPR was significantly reduced in both groups, with a large effect, and comparatively, a 1.7 times greater reduction was noted for UG patients, though not reaching statistical significance. Restricting dietary phosphorus intake without compromising dietary protein intake is essential for the management of hyperphosphatemia in HD patients [32]. Reductions in the consumption of carbonated beverages and sugar-sweetened beverages, which are energy-dense, may also explain the significant concurrent reductions in dietary energy intake for both groups (10.4% for PG vs. 12.1% for UG). Contrary to our finding, El-Khoury et al. (2020) reported that HD patients using a nutritional mobile app did not reduce their dietary phosphorus intakes but instead had an increase in dietary protein intake that resulted in a significant reduction in the PPR. In looking at changed food behaviors impacting nutritional status, serum albumin reductions occurred for both education approaches but were only significant for PG patients. However, this nutritional marker is also greatly affected by non-nutrition-related factors such as ongoing inflammation or overhydration [33].

*What was the impact of either the PMA or standard dietitian education approach on knowledge gained by the patient to moderate health behaviors?* We found that both groups benefited similarly and significantly improved their phosphorus knowledge. However, considering the five different domains of phosphate knowledge, typically covering *overall phosphate management, hyperphosphatemia consequences, phosphate binder, dietary phosphorus,* and *responsibility*, different patterns of knowledge gain emerged according to the mode of education delivery. Whilst both education delivery modes shared gains in *hyperphosphatemia consequences* and *phosphate binder,* PG patients gained additionally for *dietary phosphorus* and UG patients had specific gains in *overall phosphate management* and *responsibility.* The interactive components between food phosphate content and binder dose provided by the PMA had clearly empowered patients to discriminate the food phosphate content quantitatively according to type, portion size, and frequency [34] to enable the matching of the dose of phosphate binder [9,27].

This study did not simulate the discrepancies in the current patient education delivery practice in Malaysia that is a result of a lack of services by dedicated or visiting dietitians [14], which is typified by a single encounter upon referral without follow-up. Indeed, the Malaysian scenario reflects the global scene of dietitian shortages in chronic kidney disease care [10] and a very critical lack of dietitians specifically trained in kidney nutrition [35]. A generalist dietitian providing patient counseling, compared to a renal dietitian, would lack the critical thinking skills and knowledge [36] to enable personalized precision nutrition [37]. A concern was that initial energy and protein intake were below the recommended levels of 30–35 kcal/kg/d and 1.2 g/kg/d in this study’s patients, which reduced further with intervention. This suggests that close monitoring by a renal dietitian is critical when guiding patients in managing hyperphosphatemia to ensure adequate dietary energy and protein intakes to reduce the risk of protein–energy wasting. Further, this RCT was purposively designed to ensure that both education approaches delivered optimal nutritional knowledge, benchmarked to expert guidelines for CKD–MBD management [2]. Our study, thus, does not imply that this PMA could replace a dietitian’s role in individual patient counseling for hyperphosphatemia. A dietitian’s role in kidney disease management and its impact is beyond just the CKD–MBD surrogate markers [2] and extends to the implementation of the *Nutrition Care Process* [7]. The PMA enables generalist dietitians as well as non-dietitian healthcare professionals to narrow the gap in practice for medical nutrition therapy to enable precision nutrition-based phosphate management.

HD patients may benefit from early exposure to phosphate education using the PMA whilst waiting for a consultation with a renal dietitian. Only with intensive dietary counseling sessions delivered by renal dietitians with precision can good clinical outcomes be achieved [7,37]. This is critical, as for every 0.32 mmol/L increase of serum phosphate, the relative risk for all-cause mortality is 1.04 (1.023–1.059), *p* < 0.001 [38]. An early phosphate intervention could prevent the acceleration of hyperphosphatemia progression and reduce the risk of CKD–MBD-related mortality [39]. Thus, this innovation should narrow the healthcare practice gap, improve healthcare delivery and accessibility, and reduce the risk of mortality in the HD population in the long term.

The strengths of this study include that this RCT enabled interventions to be based on expert recommendations for CKD–MBD management, which meant that both PMA and UC patients received education according to ethical principles. Further, the patient recruitment achieved an adequate sample size to enable optimal power observations to test the hypotheses. The multicenter recruitment of patients ensured representation from government, private, and NGO dialysis centers, whilst cluster randomization for the two treatment arms prevented the cross-contamination of patients. 

Our studies also had some limitations. First, the study was only designed for three months, with only one follow-up at the end. Therefore, future studies should examine the efficacy of the PMA for a longer period, such as for 12 months. Second, the compliance measure for phosphate binder use relied on self-reporting by the patients using the Morisky score. A more accurate measure would be pill counting. Additionally, monitoring the frequency of PMA usage was not executed for this study but should be accommodated with the implementation of the PMA into clinical practice. Additionally, this study did not look into the causes of ESKD and vascular access. The study only included patients who maintained 12-h HD treatments with the same blood flow (Qb) and dialysate flow (Qd) during pre- and post-interventions. The low eligibility for study inclusion could also limit the generalization of the study findings. Lastly, the age profile of the recruited patients favored the 45 to 54 years old range whereas the majority of adult HD patients in Malaysia are in the 55 to 64 years old range (Wong 2018). However, we note that younger patients are more receptive to smartphones and mobile app use, as shown in an earlier survey [18].

## 5. Conclusions

This RCT has shown that targeted management for hyperphosphatemia can be achieved through a mobile application, not differing from dietitian management, with an additional benefit of titrating binder doses according to patient meal selections. This PMA has the potential to be optimized for HD centers without a dedicated or visiting dietitian service.

## Figures and Tables

**Table 1:** Patients’ baseline characteristics and biochemical parameters (*n* = 66).

Parameters	PG (*n* = 33)		UG (*n* = 33)		Between-Group *p*-Value
	Mean ± SD	*n* (%)	Mean ± SD	*n* (%)	
Age (year)	47.5 ± 15.3		49.15 ± 13.63		0.636 ^a^
Gender					0.805 ^b^
Male		15 (45.5)		16 (48.5)	
Female		18 (54.5)		17 (51.5)	
Ethnicity					0.356 ^b^
Malay		18 (54.5)		15 (45.5)	
Chinese		11 (33.3)		13 (39.4)	
Indian		2 (6.1)		5 (15.2)	
Others		2 (6.1)		0 (0.0)	
Marital status					0.145 ^b^
Married		6 (18.2)		10 (30.3)	
Single		27 (81.8)		21 (63.6)	
Divorced		0 (0.0)		2 (6.1)	
Education level					0.087 ^b^
Diploma/Degree/Higher		3 (9.1)		8 (24.2)	
Secondary		12 (36.4)		15 (45.5)	
Primary		18 (54.5)		10 (30.3)	
Monthly household income					0.158 ^b^
Less than RM 500		15 (45.5)		15 (45.5)	
RM 501–1000		4 (12.1)		6 (18.2)	
RM 1001–2000		1 (3.0)		5 (15.2)	
RM 2001–3000		3 (9.1)		4 (12.1)	
RM 3001–4000		5 (15.2)		0 (0.0)	
RM 4001–5000		2 (6.1)		2 (6.1)	
More than RM5000		3 (9.1)		1 (3.0)	
Employment					0.468 ^b^
Retired		10 (30.3)		11 (33.3)	
Employed for wages		5 (15.2)		5 (15.2)	
Self-employed		3 (9.1)		0 (0.0)	
Housewife		9 (27.3)		8 (24.2)	
Out of work		4 (12.1)		3 (9.1)	
Student		1 (3.0)		1 (3.0)	
Unable to work		1 (3.0)		5 (15.2)	
Smartphone OS					1.000 ^c^
Android		31 (93.9)		30 (90.9)	
Apple iOS		2 (6.1)		3 (9.1)	
Body mass index (kg/m^2^)	22.4 ± 4.1		23.9 ± 4.1		0.152 ^a^
^ꝉ^ HD vintage (month)	78 (119)		49 (52)		0.251 ^d^
Kt/v (baseline)	1.68 ± 0.30		1.69 ± 0.28		0.888 ^a^
Kt/v (3-month)	1.70 ± 0.28		1.71 ± 0.27		0.844 ^a^
Phosphate binder					0.311 ^b^
Calcium carbonate		28 (84.8)		29 (87.9)	
Sevelamer carbonate		1 (3.0)		3 (9.1)	
Lanthanum carbonate		4 (12.1)		1 (3.0)	
Activated Vitamin D					0.786 ^b^
Prescribed		10 (30.3)		9 (27.3)	
Not prescribed		23 (69.7)		24 (72.7)	
Calcimimetic					0.492 ^b^
Prescribed		2 (6.1)		0 (0.0)	
Not prescribed		31 (93.9)		33 (100.0)	
Comorbidities					
Diabetes mellitus		6 (18.2)		13 (39)	0.057 ^b^
Hypertension		22 (66.7)		25 (75.8)	0.415 ^b^
Dyslipidemia		12 (36.4)		11 (33.3)	0.796 ^b^
Heart disease		3 (9.1)		2 (6.1)	1.000 ^c^
Anemia*		9 (27.3)		5 (15.2)	0.228 ^b^
Biochemical Parameters					
Phosphorus (mmol/L)	2.34 ± 0.34		2.17 ± 0.34		0.053 ^a^
Calcium, corrected (mmol/L)	2.33 ± 0.23		2.23 ± 0.28		0.144 ^a^
iPTH ^ꝉ^ (pmol/L)	24.23 (18.81)		26.25 (15.89)		0.737 ^d^
ALP ^ꝉ^ (U/L)	150.16 (102.38)		161.38 (139.55)		0.720 ^a^
Albumin (g/L)	42.2 ± 3.3		41.1 ± 3.6		0.226 ^a^
Total phosphate knowledge score	9.6 ± 3.9		9.2± 4.1		0.690
Overall phosphate management	1.4 ± 1.0		1.1 ± 1.0		0.191
Hyperphosphatemia consequences	1.5 ± 1.0		1.6 ± 1.0		0.807
Phosphate binder	2.4 ± 1.2		2.2 ± 1.1		0.528
Dietary phosphorus	3.4 ± 1.8		3.8 ± 1.8		0.455
Responsibility	1.0 ± 0.2		0.7 ± 0.5		0.001
Total MMAS-4 score	2.1 ± 1.1		2.1 ± 1.1		1.000
Dietary intakes					
Energy (kcal/day)	1623 ± 318		1609 ± 412		0.889
Energy (kcal/kg/day)	29.1 ± 6.3		27.6 ± 7.7		0.459
Protein (g/day)	60.9 ± 15.2		60.2 ± 20.8		0.895
Protein (g/kg/day)	1.1 ± 0.3		1.0 ± 0.4		0.517
Phosphorus (mg/day)	817 ± 280		798 ± 267		0.804
PPR (mg/g)	13.5 ± 3.8		13.7 ± 3.7		0.878

^ꝉ^ Values are expressed as median (interquartile range). ^a^ Independent-samples t-test, ^b^ Pearson’s chi-squared test, ^c^ Fisher’s exact test, ^d^ Mann–Whitney test. Abbreviation: BMI, body mass index; HD, hemodialysis; iPTH, intact parathyroid hormone; Kt/V, dialysis treatment adequacy index; OS, operating system; PG, PMA group; PPR, phosphorus-to-protein ratio; UG, usual care group. * Anemia = serum hemoglobin (Hb) level < 11 g/dL.

**Table 2:** Effects of intervention on biochemical parameters.

Parameters	PG (*n =* 33)					UG (*n =* 33)					Between-Group Change *p*-Value ^b^
	Before	After	Change	Cohen’s *d* (95%CI)	Within-Group *p*-Value ^a^	Before	After	Change	Cohen’s *d* (95%CI)	Within-Group *p*-Value ^a^	
Phosphorus (mmol/L)	2.34 ± 0.34	2.08 ± 0.41	−0.25 ± 0.42	0.61 (0.25–0.97)	**0.001**	2.17 ± 0.34	1.95 ± 0.50	−0.23 ± 0.33	0.69 (0.32–1.06)	**<0.001**	0.780
Calcium, corrected (mmol/L)	2.33 ± 0.23	2.30 ± 0.25	−0.03 ± 0.13	0.19 (0.00–0.52)	0.286	2.23 ± 0.28	2.33 ± 0.25	0.09 ± 0.20	0.46 (0.10–0.82)	**0.013**	**0.007**
iPTH ^ꝉ^ (pmol/L)	24.23 (18.81)	23.83 (19.02)	0.40 ± 7.71	0.05 (0.00–0.34)	0.815 ^d^	26.25 (15.89)	23.39 (13.37)	2.86 ± 6.29	0.45 (0.00–0.97)	0.100 ^d^	0.317 ^c^
Albumin (g/L)	42.2 ± 3.3	41.1 ± 3.7	−1.1 ± 2.7	0.42 (0.05–0.78)	**0.029**	41.1 ± 3.6	40.5 ± 3.5	−0.58 ± 3.52	0.16 (0.00–0.49)	0.354	0.484

Values are expressed as mean ± SD except where indicated. Values in bold indicate *p*-value <0.05. ^ꝉ^ Data are presented as median (interquartile range). ^a^ Paired-samples *t*-test, ^b^ independent-samples *t*-test, ^c^ Mann–Whitney test, ^d^ Wilcoxon signed-rank test. Abbreviation: iPTH, intact parathyroid hormone; PG, PMA group; UG, usual care group.

**Table 3:** Effects of intervention on phosphorus knowledge score, medication adherence, and dietary intake.

Parameters	PG (*n =* 33)					UG (*n =* 33)					Between-Group Change *p*-Value ^b^
	Before	After	Change	Cohen’s *d* (95%CI)	Within-Group *p*-Value ^a^	Before	After	Change	Cohen’s *d* (95%CI)	Within-Group *p*-Value ^a^	
Phosphorus Knowledge Score											
Total knowledge score	9.6 ± 3.9	11.8 ± 2.5	2.2 ± 3.4	0.64 (0.26–1.01)	**0.001**	9.2± 4.1	11.7 ± 3.3	2.5 ± 4.5	0.56 (0.18–0.92)	**0.003**	0.758
Overall phosphate management	1.4 ± 1.0	1.5 ± 0.9	0.2 ±1.0	-	0.406	1.1 ± 1.0	1.6 ±0.9	0.5 ± 1.2	-	**0.019**	0.192
Hyperphosphatemia consequences	1.5 ± 1.0	1.9 ± 0.9	0.4 ± 0.8	-	**0.016**	1.6 ± 1.0	1.9 ±0.9	0.3 ± 0.9	-	**0.032**	0.884
Phosphate binder	2.4 ± 1.2	3.1 ± 1.0	0.8 ± 1.1	-	**<0.001**	2.2 ± 1.1	2.9 ±1.1	0.7 ± 1.5	-	**0.011**	0.852
Dietary phosphorus	3.4 ±1.8	4.3 ± 1.5	0.9 ± 1.9	-	**0.014**	3.8 ± 1.8	4.5 ± 1.8	0.7 ± 2.5	-	0.118	0.742
Responsibility	1.0 ± 0.2	1.0 ± 0	0 ± 0.2	-	0.325	0.7 ± 0.5	0.9 ± 0.3	0.2 ± 0.6	-	**0.018**	0.042
Medication Adherence											
Total MMAS-4 score	2.1 ± 1.1	3.2 ± 1.1	1.1 ± 1.2	0.90 (0.49–1.30)	**<0.001**	2.1 ± 1.1	2.9 ± 1.3	0.8 ± 1.5	0.50 (0.14–0.86)	**0.007**	0.324
Adherence	4(12.1)	20(60.6)	↑ 16(48.5)		**<0.001 ^a^**	5(15.2)	16 (48.5)	↑ 11(33.3)		**0.001 ^a^**	0.317 ^c^
Non-adherence	29(87.9)	13(39.4)				28(84.8)	17(51.5)				
Dietary Intakes											
Energy (kcal/day)	1623 ± 318	1433 ± 389	−191 ± 306	0.62 (0.21–1.04)	**0.005**	1609 ± 412	1392 ± 384	−217 ± 274	0.79 (0.34–1.24)	**0.001**	0.755
Energy (kcal/kg/day)	29.1 ± 6.3	25.6 ± 6.1	−3.4 ± 5.8	0.60 (0.19–1.01)	**0.007**	27.6 ± 7.7	23.9 ± 7.1	−3.7 ± 4.9	0.75 (0.30–1.19)	**0.001**	0.868
Protein (g/day)	60.9 ± 15.2	58.3 ± 17.2	−2.6 ± 18.8	0.14 (0.00–0.52)	0.492	60.2 ± 20.8	57.5 ± 17.2	−2.7 ± 20.3	0.13 (0.00–0.52)	0.520	0.989
Protein (g/kg/day)	1.1 ± 0.3	1.0 ± 0.3	−0.0 ± 0.3	0.15 (0.00–0.52)	0.471	1.0 ± 0.4	1.0 ± 0.3	−0.0 ± 0.4	0.11 (0.00–0.49)	0.601	0.929
Phosphorus (mg/day)	817 ± 280	629 ± 212	−188 ± 161	1.17 (0.65–1.67)	**<0.001**	798 ± 267	572 ± 199	−226 ± 193	1.17 (0.64–1.68)	**<0.001**	0.462
PPR (mg/g)	13.5 ± 3.8	11.6 ± 4.6	−1.9 ± 3.1	0.62 (0.20–1.04)	**0.005**	13.7 ± 3.7	10.0 ± 2.0	−3.7 ± 3.5	1.07 (0.56–1.57)	**<0.001**	0.066

Values are expressed as mean ± SD. Values in bold indicate *P*-value <0.05. ^a^ Paired-samples *t*-test, ^b^ independent-samples *t*-test, ^c^ Chi-square test. Abbreviation: PG, PMA group; PPR, phosphorus-to-protein ratio; UG, usual care group. Symbol “↑” indicates increase.

## Data Availability

The datasets generated and analyzed for the current study are available from the corresponding author, T.K., upon reasonable request.
